# The Strengthening the Reporting of Observational Studies in Epidemiology (STROBE) Statement: Guidelines for Reporting Observational Studies

**DOI:** 10.1371/journal.pmed.0040296

**Published:** 2007-10-16

**Authors:** Erik von Elm, Douglas G Altman, Matthias Egger, Stuart J Pocock, Peter C Gøtzsche, Jan P Vandenbroucke, for the STROBE Initiative

**Affiliations:** 1 Institute of Social and Preventive Medicine (ISPM), University of Bern, Bern, Switzerland; 2 Centre for Statistics in Medicine, University of Oxford, Oxford, United Kingdom; 3 Department of Social Medicine, University of Bristol, Bristol, United Kingdom; 4 London School of Hygiene and Tropical Medicine, University of London, London, United Kingdom; 5 Nordic Cochrane Centre, Copenhagen, Denmark; 6 Department of Clinical Epidemiology, Leiden University Hospital, Leiden, The Netherlands

## Abstract

Much biomedical research is observational. The reporting of such research is often inadequate, which hampers the assessment of its strengths and weaknesses and of a study's generalisability. The Strengthening the Reporting of Observational Studies in Epidemiology (STROBE) Initiative developed recommendations on what should be included in an accurate and complete report of an observational study. We defined the scope of the recommendations to cover three main study designs: cohort, case-control, and cross-sectional studies. We convened a 2-day workshop in September 2004, with methodologists, researchers, and journal editors to draft a checklist of items. This list was subsequently revised during several meetings of the coordinating group and in e-mail discussions with the larger group of STROBE contributors, taking into account empirical evidence and methodological considerations. The workshop and the subsequent iterative process of consultation and revision resulted in a checklist of 22 items (the STROBE Statement) that relate to the title, abstract, introduction, methods, results, and discussion sections of articles. 18 items are common to all three study designs and four are specific for cohort, case-control, or cross-sectional studies. A detailed Explanation and Elaboration document is published separately and is freely available on the Web sites of *PLoS Medicine, Annals of Internal Medicine,* and *Epidemiology.* We hope that the STROBE Statement will contribute to improving the quality of reporting of observational studies.

## Abstract 2

This paper describes the recommendations of The Strengthening the Reporting of Observational Studies in Epidemiology (STROBE) Initiative on what should be included in an accurate and complete report of an observational study.

## Introduction

Many questions in medical research are investigated in observational studies [1]. Much of the research into the cause of diseases relies on cohort, case-control, or cross-sectional studies. Observational studies also have a role in research into the benefits and harms of medical interventions [2]. Randomised trials cannot answer all important questions about a given intervention. For example, observational studies are more suitable to detect rare or late adverse effects of treatments, and are more likely to provide an indication of what is achieved in daily medical practice [3].

Research should be reported transparently so that readers can follow what was planned, what was done, what was found, and what conclusions were drawn. The credibility of research depends on a critical assessment by others of the strengths and weaknesses in study design, conduct, and analysis. Transparent reporting is also needed to judge whether and how results can be included in systematic reviews [4,5]. However, in published observational research important information is often missing or unclear. An analysis of epidemiological studies published in general medical and specialist journals found that the rationale behind the choice of potential confounding variables was often not reported [6]. Only few reports of case-control studies in psychiatry explained the methods used to identify cases and controls [7]. In a survey of longitudinal studies in stroke research, 17 of 49 articles (35%) did not specify the eligibility criteria [8]. Others have argued that without sufficient clarity of reporting, the benefits of research might be achieved more slowly [9], and that there is a need for guidance in reporting observational studies [10,11].

Recommendations on the reporting of research can improve reporting quality. The Consolidated Standards of Reporting Trials (CONSORT) Statement was developed in 1996 and revised 5 years later [12]. Many medical journals supported this initiative [13], which has helped to improve the quality of reports of randomised trials [14,15]. Similar initiatives have followed for other research areas—e.g., for the reporting of meta-analyses of randomised trials [16] or diagnostic studies [17]. We established a network of methodologists, researchers, and journal editors to develop recommendations for the reporting of observational research: the Strengthening the Reporting of Observational Studies in Epidemiology (STROBE) Statement.

## Aims and Use of the STROBE Statement

The STROBE Statement is a checklist of items that should be addressed in articles reporting on the 3 main study designs of analytical epidemiology: cohort, case-control, and cross-sectional studies. The intention is solely to provide guidance on how to report observational research well: these recommendations are not prescriptions for designing or conducting studies. Also, while clarity of reporting is a prerequisite to evaluation, the checklist is not an instrument to evaluate the quality of observational research.

Here we present the STROBE Statement and explain how it was developed. In a detailed companion paper, the Explanation and Elaboration article [18–20], we justify the inclusion of the different checklist items and give methodological background and published examples of what we consider transparent reporting. We strongly recommend using the STROBE checklist in conjunction with the explanatory article, which is available freely on the Web sites of *PLoS Medicine* (http://www.plosmedicine.org/), *Annals of Internal Medicine* (http://www.annals.org/), and *Epidemiology* (http://www.epidem.com/).

## Development of the STROBE Statement

We established the STROBE Initiative in 2004, obtained funding for a workshop and set up a Web site (http://www.strobe-statement.org/). We searched textbooks, bibliographic databases, reference lists, and personal files for relevant material, including previous recommendations, empirical studies of reporting and articles describing relevant methodological research. Because observational research makes use of many different study designs, we felt that the scope of STROBE had to be clearly defined early on. We decided to focus on the 3 study designs that are used most widely in analytical observational research: cohort, case-control, and cross-sectional studies.

We organised a 2-day workshop in Bristol, UK, in September 2004. 23 individuals attended this meeting, including editorial staff from *Annals of Internal Medicine*, *BMJ*, *Bulletin of the World Health Organization*, *International Journal of Epidemiology*, *JAMA*, *Preventive Medicine*, and *The Lancet,* as well as epidemiologists, methodologists, statisticians, and practitioners from Europe and North America. Written contributions were sought from 10 other individuals who declared an interest in contributing to STROBE, but could not attend. Three working groups identified items deemed to be important to include in checklists for each type of study. A provisional list of items prepared in advance (available from our Web site) was used to facilitate discussions. The 3 draft checklists were then discussed by all participants and, where possible, items were revised to make them applicable to all three study designs. In a final plenary session, the group decided on the strategy for finalizing and disseminating the STROBE Statement.

After the workshop we drafted a combined checklist including all three designs and made it available on our Web site. We invited participants and additional scientists and editors to comment on this draft checklist. We subsequently published 3 revisions on the Web site, and 2 summaries of comments received and changes made. During this process the coordinating group (i.e., the authors of the present paper) met on eight occasions for 1 or 2 days and held several telephone conferences to revise the checklist and to prepare the present paper and the Explanation and Elaboration paper [18–20]. The coordinating group invited 3 additional co-authors with methodological and editorial expertise to help write the Explanation and Elaboration paper, and sought feedback from more than 30 people, who are listed at the end of this paper. We allowed several weeks for comments on subsequent drafts of the paper and reminded collaborators about deadlines by e-mail.

## STROBE Components

The STROBE Statement is a checklist of 22 items that we consider essential for good reporting of observational studies (Table 1). These items relate to the article's title and abstract (item 1), the introduction (items 2 and 3), methods (items 4–12), results (items 13–17) and discussion sections (items 18–21), and other information (item 22 on funding). 18 items are common to all three designs, while four (items 6, 12, 14, and 15) are design-specific, with different versions for all or part of the item. For some items (indicated by asterisks), information should be given separately for cases and controls in case-control studies, or exposed and unexposed groups in cohort and cross-sectional studies. Although presented here as a single checklist, separate checklists are available for each of the 3 study designs on the STROBE Web site.

**Table 1:**
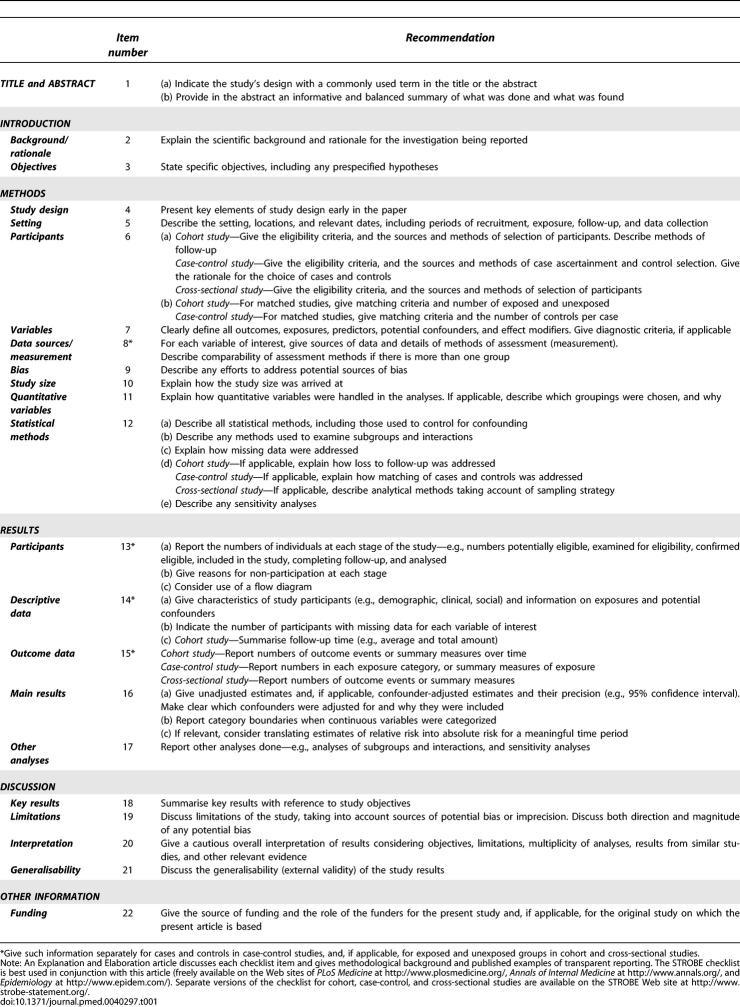
The STROBE Statement—Checklist of Items That Should Be Addressed in Reports of Observational Studies

## Implications and Limitations

The STROBE Statement was developed to assist authors when writing up analytical observational studies, to support editors and reviewers when considering such articles for publication, and to help readers when critically appraising published articles. We developed the checklist through an open process, taking into account the experience gained with previous initiatives, in particular CONSORT. We reviewed the relevant empirical evidence as well as methodological work, and subjected consecutive drafts to an extensive iterative process of consultation. The checklist presented here is thus based on input from a large number of individuals with diverse backgrounds and perspectives. The comprehensive explanatory article [18–20], which is intended for use alongside the checklist, also benefited greatly from this consultation process.

Observational studies serve a wide range of purposes, on a continuum from the discovery of new findings to the confirmation or refutation of previous findings [18–20]. Some studies are essentially exploratory and raise interesting hypotheses. Others pursue clearly defined hypotheses in available data. In yet another type of studies, the collection of new data is planned carefully on the basis of an existing hypothesis. We believe the present checklist can be useful for all these studies, since the readers always need to know what was planned (and what was not), what was done, what was found, and what the results mean. We acknowledge that STROBE is currently limited to three main observational study designs. We would welcome extensions that adapt the checklist to other designs—e.g., case-crossover studies or ecological studies—and also to specific topic areas. Four extensions are now available for the CONSORT statement [21–24]. A first extension to STROBE is underway for gene-disease association studies: the STROBE Extension to Genetic Association studies (STREGA) initiative [25]. We ask those who aim to develop extensions of the STROBE Statement to contact the coordinating group first to avoid duplication of effort.

The STROBE Statement should not be interpreted as an attempt to prescribe the reporting of observational research in a rigid format. The checklist items should be addressed in sufficient detail and with clarity somewhere in an article, but the order and format for presenting information depends on author preferences, journal style, and the traditions of the research field. For instance, we discuss the reporting of results under a number of separate items, while recognizing that authors might address several items within a single section of text or in a table. Also, item 22, on the source of funding and the role of funders, could be addressed in an appendix or in the methods section of the article. We do not aim at standardising reporting. Authors of randomised clinical trials were asked by an editor of a specialist medical journal to “CONSORT” their manuscripts on submission [26]. We believe that manuscripts should not be “STROBEd”, in the sense of regulating style or terminology. We encourage authors to use narrative elements, including the description of illustrative cases, to complement the essential information about their study, and to make their articles an interesting read [27].

We emphasise that the STROBE Statement was not developed as a tool for assessing the quality of published observational research. Such instruments have been developed by other groups and were the subject of a recent systematic review [28]. In the Explanation and Elaboration paper, we used several examples of good reporting from studies whose results were not confirmed in further research – the important feature was the good reporting, not whether the research was of good quality. However, if STROBE is adopted by authors and journals, issues such as confounding, bias, and generalisability could become more transparent, which might help temper the over-enthusiastic reporting of new findings in the scientific community and popular media [29], and improve the methodology of studies in the long term. Better reporting may also help to have more informed decisions about when new studies are needed, and what they should address.

We did not undertake a comprehensive systematic review for each of the checklist items and sub-items, or do our own research to fill gaps in the evidence base. Further, although no one was excluded from the process, the composition of the group of contributors was influenced by existing networks and was not representative in terms of geography (it was dominated by contributors from Europe and North America) and probably was not representative in terms of research interests and disciplines. We stress that STROBE and other recommendations on the reporting of research should be seen as evolving documents that require continual assessment, refinement, and, if necessary, change. We welcome suggestions for the further dissemination of STROBE—e.g., by re-publication of the present article in specialist journals and in journals published in other languages. Groups or individuals who intend to translate the checklist to other languages should consult the coordinating group beforehand. We will revise the checklist in the future, taking into account comments, criticism, new evidence, and experience from its use. We invite readers to submit their comments via the STROBE Web site (http://www.strobe-statement.org/).

## Abbreviations

CONSORT: Consolidated Standards of Reporting Trials
STREGA: STROBE Extension to Genetic Association Studies
STROBE: Strengthening the Reporting of Observational Studies in Epidemiology
